# Antimicrobial Peptide–Polymer Conjugates

**DOI:** 10.3390/pharmaceutics14102171

**Published:** 2022-10-12

**Authors:** Martijn Riool, Viorica Patrulea, Cláudia Monteiro

**Affiliations:** 1 Department of Medical Microbiology and Infection Prevention, Amsterdam Institute for Infection and Immunity, Amsterdam UMC, University of Amsterdam, Meibergdreef 9, 1105 AZ Amsterdam, The Netherlands; 2 Institute of Biomedical Engineering, Department of Engineering Science, University of Oxford, Oxford OX3 7DQ, UK; 3 I3S—Instituto de Investigação e Inovação em Saúde, Universidade do Porto, Rua Alfredo Allen, 208, 4200-135 Porto, Portugal; 4 INEB—Instituto de Engenharia Biomédica, Universidade do Porto, Rua Alfredo Allen, 208, 4200-135 Porto, Portugal

The global health threat imposed by the fast spread of antibiotic-resistant bacteria is directing research not only towards the discovery of new antibacterial molecules but also to the repurposing of old drugs, while improving their efficiency and safety. Antimicrobial peptides (AMPs), also known as host defence peptides (HDPs), are expressed by all multicellular organisms and have direct and indirect antimicrobial actions, i.e., eliminating bacteria by membrane disruption, inhibiting and eradicating biofilms through inhibition of signalling pathways, and modulating the innate immune response. AMPs have been extensively studied in recent decades and are widely recognized as promising alternatives to conventional antibiotics. Despite the large number of candidates as evidenced by the number of entries (22,480) in the DRAMP database (Data Repository for Antimicrobial Peptides), very few have reached the clinic [1]. This is mainly the result of the low bioavailability of AMPs, which leads to fast clearance and hinders AMPs from reaching their therapeutic target. In fact, AMPs are susceptible to enzymatic degradation, are poorly absorbed at the intestinal mucosa, and are affected by opsonization and binding to serum proteins when intravenously administered.

In this regard, AMP mimics, conjugates, and delivery systems are being developed to improve AMPs’ bioactivity while reducing the undesirable side effects. This Special Issue is mainly composed of reviews and represents a valuable information resource of the latest developments of AMP-based therapies.

Etayash and Hancock summarize the recent developments in (i) amphiphilic antimicrobial polymers, structural mimics of AMPs, and (ii) polymeric biomaterials functionalized with brush-tethered AMPs to fight infections [2]. Both strategies have their benefits and limitations, and AMP-mimicking polymers, unlike AMPs, are stable and easy to produce. These polymers have the same advantages as AMPs, e.g., a low propensity to develop resistance and broad-spectrum, antibiofilm, and immune-modulatory activities. In addition, some of these polymers have demonstrated antifungal activity, are more resistant to degradation, and, in general, large-scale production can be adapted to easy and cost-effective methods. Polymers, as observed for AMPs, often possess significant toxicity to eukaryotic cells, probably related to their non-selective mode of action. However, attempts to reduce the cytotoxicity often also leads to reduced antimicrobial activity, a general observation also seen for AMPs. Moreover, polymers frequently have high molecular weights and are therefore difficult to solubilize and act slower. Etayash and Hancock provide an excellent overview of the structural features influencing the bioactivity and toxicity of AMPs, such as cationic and hydrophobic functional groups, the hydrophilic/hydrophobic balance, and the polymer molecular weight, which are all important elements in designing better polymer mimics of AMPs.

The second strategy described in this review is the functionalization of polymeric biomaterials with brush-tethered AMPs, used for the functionalization of implants [2]. This strategy allows larger amounts of AMPs to be grafted, increasing the local AMP concentration and consequently increasing the efficiency. The activity of the peptides is influenced by factors such as peptide sequence and spacer length, the type of surface coated, and environmental factors such as pH and location in the body (e.g., flow). Additionally, it must be considered that bacteria are caught and subsequently killed by immobilized AMPs at the surface, remain on the surface, and shield the antimicrobial activity, providing a substrate for another layer of bacteria to grow. Despite the limitations mentioned, the authors mention that these brush-tethered AMPs represent one of the most promising strategies to fight infections on implant surfaces.

Costa et al. review the potential of AMPs to fight orthopaedic- and dental-implant-related infections [3]. The authors discuss different release strategies to ensure the higher loading capacity and prolonged release of the AMPs without affecting biocompatibility, such as AMP-loaded bone cements or titanium-based release systems. Moreover, several immobilization strategies to prevent infection at later stages are covered, such as direct surface functionalization, the application of chimeric peptides and AMP-grafted polymeric coatings. The authors conclude that a combination of the release and immobilization of AMPs appears to be the best way to go forward to guarantee the prevention of the colonization of the wound during surgery, as well as biofilm formation at the implant site at later stages. One major hurdle addressed is the difficulty of translating these novel antimicrobial strategies to the clinic. Therefore, the authors highlight the importance of ensuring that in vitro validation simulates more realistic scenarios and timeframes and in vivo validation compares directly to standard care or newly developed technology.

Regarding AMP susceptibility to degradation, it has long been known that *D*-configurations of amino acids (*D*-aa) are resistant to degradation and are useful in overcoming this critical limitation; however, the mechanism of the bacterial killing of AMPs containing *D*-aa has not been fully elucidated yet. Bellotto et al. summarize the advantages and disadvantages of the most recent covalently coupled *D*-aa AMPs (e.g., daptomycin, gramicidin S, polymyxins, and vancomycin) to polymers as drug delivery vehicles [4]. They highlight the strategy of preventing and eradicating *Staphylococcus aureus* biofilm on titanium implants by conjugating daptomycin to a mussel-inspired, tetrazine-functionalized coating [5]. This is of major importance for the treatment of implant-associated methicillin-resistant *S. aureus* (MRSA) infections, particularly in surgical settings. An alternative strategy involves gramicidin S, a potent AMP, which can be covalently coupled to potassium acyltrifluoroborates (KATs) polymers via a chemoselective atom-transfer radical polymerization (ATRP) and reversible addition–fragmentation chain transfer (RAFT) reaction. The gramicidin-polymer-based delivery system is biocompatible and provides a controlled release of AMPs, showing activity against the Gram-positive *Bacillus subtilis* upon irradiation with UV light [6]. This approach could be used in wound bandages to eradicate bacteria, simply exposing them to UV. The authors also outline that despite the efficient coupling of colistin to dextrin, the amylase-degraded dextrin–colistin conjugate is not the most efficient, mainly due to residual colistin which binds to the linker, affecting the AMPs’ bioactivity [7]. It is also extremely important to know the limitations of the systems in order to provide focus to research efforts. On the other hand, the PEGylation of colistin shows efficiency against *Acinetobacter baumannii* and *Pseudomonas aeruginosa* [8]. Interestingly, this group points out that both polymyxin B and vancomycin can be efficient against *Escherichia coli* and *S. aureus* if both dextran and PEG are used and integrated into wound dressings [9]. This is of particular interest for wound-healing therapies.

Gent et al. focus on the most promising lipid and polymeric AMP delivery systems and coatings for bacteria and biofilm eradication. One of the most efficient AMP delivery systems, the liposome delivery system, has been determined to be efficient in the delivery of a variety of AMPs, including colistin, vancomycin, LL-37, indolicidin, and polymyxin B. These systems have been successfully used against both Gram-positive and Gram-negative bacteria. Niosomes, which are more stable and cheaper than liposomes, are listed as efficient tools in vitro against *P. aeruginosa* and ex vivo in liver cells for the safe delivery of polymyxin B [10]. Additionally, solid lipid nanoparticles are considered as promising alternatives to deliver a variety of AMPs, including lacticin 3147, polymyxin B, and LL-37 [11]. Although micellar systems are seldom found in the literature, they are considered as potential tools for AMP delivery. Aurein-derived AMPs can be covalently coupled to PEGylated micelles without hindering their native activity. Interestingly, they can reach 9- and 510-fold higher antibacterial activity than the parent aurein peptide against *S. aureus* biofilm in vitro and *S. aureus* in a murine cutaneous abscess model, respectively [12]. Importantly, the authors mention two important challenges when bringing AMPs into clinics: (i) a lack of standardized protocols under biorelevant conditions when testing the antibacterial activity and (ii) a lack of shelf-stable formulations.

In conclusion, several challenges remain in the application of AMP conjugates and delivery systems in the clinic. The specificities of each AMP conjugate/delivery system make translation more difficult. However, this will be a subject of interest in the coming decades and will require efforts regarding regulatory policies and access to clinical trials.
